# Trends in the pervasiveness of type 2 diabetes, impaired fasting glucose and co-morbidities during an 8-year-follow-up of nationwide Korean population

**DOI:** 10.1038/srep46656

**Published:** 2017-04-20

**Authors:** Junghyun Noh, Kyung-Do Han, Seung-Hyun Ko, Kyung Soo Ko, Cheol-Young Park

**Affiliations:** 1 Department of Internal Medicine, Inje University Ilsan Paik Hospital, Goyang, Republic of Korea; 2 Department of Medical Statistics, Catholic University College of Medicine, Seoul, South Korea; 3 Department of Internal Medicine, St. Vincent’s Hospital, The Catholic University of Korea, Seoul, Korea; 4 Department of Internal Medicine, Cardiovascular and Metabolic Disease Center, Inje University Sanggye Paik Hospital, Seoul, Republic of Korea; 5 Department of Internal Medicine, Kangbuk Samsung Hospital, Sungkyunkwan University School of Medicine, Seoul, Republic of Korea

## Abstract

The prevalence and incidence of type 2 diabetes, impaired fasting glucose, and co-morbidities from 2006 to 2013 in the population aged ≥30 years were estimated using the Korean NHIS database. The prevalence of type 2 diabetes increased 0.2–0.5% annually, from 5.6% in 2006 to 8% in 2013. The prevalence of type 2 diabetes was higher in men than in women and increased with age. The incidence of type 2 diabetes was 0.81% in 2013 and was 1.4 times higher in men than in women and increased with age. An overall decrease in the incidence rate occurred from 2006 to 2013(from 0.95 to 0.81%), which was mirrored in all age groups except the 30–39-year-old group. The prevalence of IFG was 25% in 2013. The prevalence of hypertension(62.5 vs 16.9%) and dyslipidemia(49.5 vs 9.7%) were more prevalent in patients with type 2 diabetes compared to non-diabetic cases. This study shows that type 2 diabetes is both common and increasing and that one-quarter of the Korean adult population has IFG. We also confirmed that the prevalence of hypertension and dyslipidemia are 3.7-fold and 5.1-fold higher, respectively, in diabetic patients than in non-diabetic adults.

Diabetes, which causes damage to many organ systems, is a considerable health challenge and economic burden for society, as well as for the individual^1^,^2^. Epidemiological studies that include the prevalence of type 2 diabetes, prediabetes, and concomitant co-morbidities have a pronounced impact on proper disease management, prevention programs, and estimations of the burden of these diseases^3^. Type 2 diabetes has rapidly increased in prevalence in Asian populations, and the International Diabetes Federation estimates that among the 382 million people affected with diabetes, over 200 million come from Asia^4^,^5^,^6^. In China, the most recent national study found that 11.6% of the population has diabetes and 15.5% has prediabetes^7^. In Taiwan, the prevalence rate of diabetes rose from 4.9% in 1985 to 8.3% in 2007^8^,^9^. The prevalence of probable diabetes in Japan has also increased from 6.8 million to 13.2 million between 1997 and 2007^10^.

The prevalence of type 2 diabetes is increasing in Korea as well. Among adults aged ≥30 years, diabetes prevalence has increased from 8.9%, to 9.1%, to 9.9% and to 11.1% in 2001, 2005, 2007–2009, and 2014, respectively, according to the Korean National Health and Nutrition Examination Surveys(KNHANES)^11^,^12^,^13^. KNHANES is a cross-sectional health surveillance program conducted by the Korea Institute for Health and Social Affairs. Although it is a nationally representative survey, KNHANES is a sampling survey, not a complete enumeration.

The National Health Insurance Service(NHIS) in Korea is a single-payer program and is mandatory for all residents in Korea. The Health Insurance Review and Assessment(HIRA) database, operated under NHIS, includes records of billing and claims. NHIS also provides routine annual preventive health check-ups for adults aged ≥40 years or employees who are insured^14^. Recently, the NHIS signed an agreement with the Korean Diabetes Association(KDA) to provide open access to the databases for the benefit of Korean subjects with diabetes.

The present study aims to analyze the prevalence and incidence of type 2 diabetes, prediabetes, and co-morbidities in Korea from 2006 to 2013 in the population aged ≥30 years using the Korean NHIS database.

## Results

### Prevalence and incidence of type 2 diabetes

From the NHIS database, the source population aged ≥30 years and total number of type 2 diabetes cases were 29.3 million and 1,655,495, respectively, in 2006 and 33.9 million and 2,720,777, respectively, in 2013. The prevalence of type 2 diabetes, which has been increasing annually by 0.2–0.5%, was 5.6% in 2006 and 8% in 2013, which represents an increase of 42.9% in the annual prevalence(Table 1). The prevalence of type 2 diabetes was 1.2 times higher in men than in women and increased with age from 0.9% in ages 30–39, to 3.5% in ages 40–49, to 8.9% in ages 50–59, to 16.6% in ages 60–69, and to 21.5% in ages 70–79, with a slight decrease in the ≥80 years age group(16.7%). The mean age of the prevalent cases in 2013 was 62.9 ± 12.0 years. The annual increase in diabetes prevalence rates was largest in the ≥80 years age group, which had a 70.4% increase from 2006 to 2013(Supplementary Fig. 1). The age-standardized prevalence of type 2 diabetes varies by region. The region with the highest prevalence was Sejong city, while Jeju Island showed the lowest prevalence of type 2 diabetes. Urban and rural areas both showed a similar prevalence of type 2 diabetes(Supplementary Fig. 2).

We also analyzed the prevalence of type 2 diabetes from the preventive health check-up data. The source populations aged ≥30 years who had routine checkups from the National Preventive Health Care system were estimated at 7.2 and 10.6 million persons in 2006 and 2013, respectively. For 2013, the prevalence of type 2 diabetes, as analyzed from the preventive health check-up data, was 10.9% as opposed to 8% as analyzed from the insurance claims data. When defined using claims for prescriptions of anti-diabetic medications under diabetes codes, the prevalence rate of type 2 diabetes was 7.8% compared to 3.1% when defined according to fasting plasma glucose levels without claims under ICD-10 codes E11–14 that suggested undiagnosed diabetes in 2013(Supplementary Fig. 3). The pattern of annual increase in diabetes prevalence rates from the preventive health check-up data was the same as from the insurance claims data(Table 1 and Supplementary Fig. 3). However, when defined as a fasting plasma glucose ≥126 mg/dL without claims under ICD-10 codes E11–14(suggestive of undiagnosed diabetes), diabetes prevalence rates decreased over time(p < 0.001, Supplementary Fig. 3).

The numbers of newly-diagnosed patients with type 2 diabetes, as analyzed from the insurance claims data, were 265,346 and 253,589 in 2006 and 2013, respectively. The overall incidence of type 2 diabetes was 0.81% in 2013, but the rate was 1.4 times higher in men than in women and increased with age from 0.25% in ages 30–39, to 0.54% in ages 40–49, to 1.03% in ages 50–59, to 1.47% in ages 60–69, and to 1.65% in ages 70–79, with a slight decrease in the ≥80 years age group(1.25%). The annual incidence of type 2 diabetes decreased over time in all age groups except 30–39, with an overall decrease of 17.3% from 2006 to 2013(Table 2).

We also analyzed the age categorized incidence of T2DM for 8 years from NHIS data. The incidence of T2DM as per 1,000 residents for 8 years was 3.0 in ages 30–39, 7.3 in ages 40–49, 12.7 in ages 50–59, 17.0 in ages 60–69, 16.5 in ages 70–79 and 9.4 in ages ≥80. The incidence of T2DM for 8 years showed similar trends in this age-period cohort analysis compare to annual incidence of T2DM.(Supplementary Table 1).

### Prevalence of impaired fasting glucose

The prevalence of impaired fasting glucose, defined as fasting plasma glucose levels of 100–125 mg/dL without a prescription for anti-diabetic medication or diabetes-associated disease codes, was 25% in 2013. In the same year, the mean age of the impaired fasting glucose cases was 52.4 ± 12.4 years. The prevalence of impaired fasting glucose increased by 16.3% from 2006 to 2013. The group aged ≥80 years showed the highest prevalence, which is 1.5 times higher than in the group aged 30–39 years(Table 3).

### Prevalence and control rates of comorbid diseases

**Figure 1:**
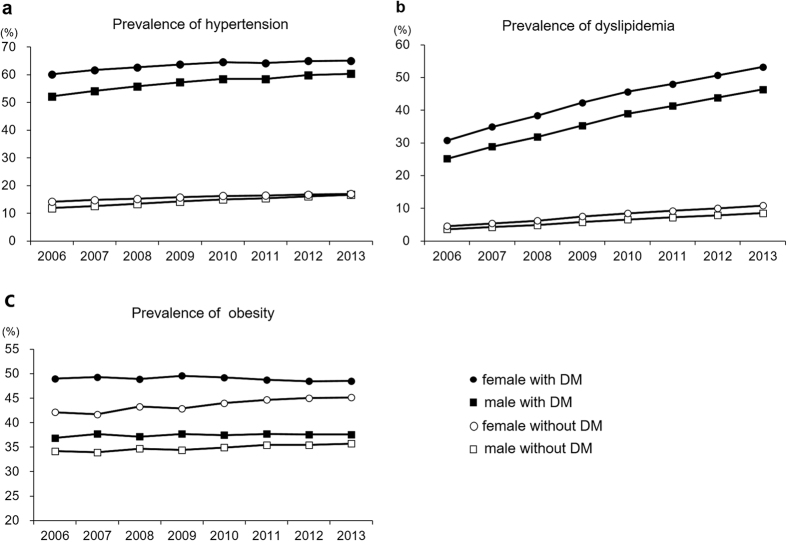
Prevalence of(**a**) hypertension,(**b**) dyslipidemia and(**c**) obesity in type 2 diabetes from 2006 to 2013.

The prevalence of hypertension, as analyzed from the insurance claims data, was 62.5% in patients with type 2 diabetes(men 60.3%, women 65%) and 16.9% in cases without diabetes(men 16.7%, women 17%), indicating that the prevalence of hypertension was notably higher in subjects with diabetes(Fig. 1). The rate of controlled hypertension, as analyzed from the preventive health check-up data, was lower in patients with type 2 diabetes(75.8%) compared to non-diabetic subjects(87.1%, *P* < 0.001) in 2013. An improvement in the rate of controlled hypertension was observed between 2006 and 2013, from 66.7% to 75.8%(*P* < 0.001, Supplementary Table 2).

The prevalence of dyslipidemia, as analyzed from the insurance claims data, was 49.5% in patients with type 2 diabetes(men 46.4%, women 53.2%), which is 5.1 times higher than in non–diabetic cases(total 9.7%, men 8.5%, women 10.8%, Fig. 1). The prevalence of dyslipidemia had an explosive increase of 78% from 2006(27.8%) to 2013(49.5%, *P* < 0.001). The rates of controlled dyslipidemia were 45.3% when defined as LDL-cholesterol < 100 mg/dL, 55.2% when defined as triglycerides <150 mg/dL, and 68.3% when defined as HDL-cholesterol ≥40 in men and ≥50 mg/dL in women. Only 18.8% of diabetes subjects met all target goals for controlled dyslipidemia.

The prevalence of obesity in 2013, as analyzed from the preventive health check-up data, was higher in women than in men in both type 2 diabetes patients(men 37.6%, women 48.5%) and non-diabetic cases(men 35.7%, women 45.2%), and higher in patients with type 2 diabetes than in non-diabetic subjects. The prevalence of obesity was stable for 8 years in both type 2 diabetic patients and non-diabetic subjects.

## Discussion

The present study provides the first representative, enumerated estimates of the prevalence and incidence of diabetes and impaired fasting glucose in Korean adults from 2006 to 2013. All data was taken from the nation-wide National Health Insurance Service shared database, which includes the insurance claims data of almost all of the Korean population and the routine preventive health check-up data for one third of the adult Korean population over 30 years of age. The overall prevalence of diabetes, as estimated from claims data, was 8.0% in 2013. An increase in the prevalence rate and decrease in the incidence rate were observed from 2006 to 2013.

The Korean prevalence rate of type 2 diabetes reported in previous studies was calculated using sample survey data such as KNHANES or regional cohort data^15^,^16^,^17^,^18^. The cross-sectional studies in various areas of Korea, including Yonchon County in 1993^17^, Jungup District in 1997^18^, and Mokdong in Seoul in 1998^19^, showed a prevalence of 7.1–8.5%. These studies were based on relatively small samples of 2520, 1108, and 774 subjects, respectively, and the data was collected in restricted areas. KNHANES, conducted by the Korea Institute for Health and Social Affairs, is a cross-sectional health surveillance system, which randomly samples throughout South Korea^13^. KNHANES showed that the prevalence of diabetes among adults aged ≥30 years in Korea was 8.9%, 9.1%, 9.9%, and 11.1% in 2001, 2005, 2007–2009, and 2014, respectively^11^,^12^,^13^. Although KNHANES is a nationally-representative survey, it is not a complete enumeration, but rather a sampling survey with 4,154–13,512 individuals used for the analysis of diabetes prevalence. Sampling surveys, including KNHANES, can have sampling errors or bias. According to KNHANES, the prevalence of diabetes in 1998 was 11.1%, which is considerably higher than the prevalence in the next several years^11^,^13^. The prevalence of diabetes in women also showed a fluctuation from 7.3%, to 8.4%, and to 7.6% in 2007, 2009, and 2011, respectively^11^,^13^. These results suggest the existence of limitations in the KNHANES database. The National Health Insurance Service shared database used in this study is the only completely-enumerated survey for all residents in Korea. For this reason, the present study is more reliable and provides more precise estimates compared to previous studies.

The analysis showed that the annual prevalence of type 2 diabetes continued to rise throughout the study period, similar to estimates from other Asian countries^4^,^5^,^6^,^7^,^8^,^9^,^10^. Despite an increase in prevalence, the annual incidence rate gradually decreased. This phenomenon may partly be explained by better diabetes care and increasing life expectancies, which is supported by the fact that the average life expectancy of Koreans increased from 79.2 years in 2006 to 81.9 years in 2013^20^. In addition, the annual increase in diabetes prevalence rates was largest in the ≥80 years age group, which also supports the conclusion that increasing life expectancies contributes to the increase of prevalence of type 2 diabetes. Furthermore, the prevalence of diabetes is 6.6 times higher in the age 70–79 group compared to the age 30–39 group, but impaired fasting glucose is only 1.5 times higher in the same comparison, which supports the prediction that there will be an increased prevalence of type 2 diabetes in the future.

Sejong city, South Korea’s de facto administrative capital city created and opened in 2012, showed the highest age-standardized prevalence of type 2 diabetes in 2013. Because of regional characteristics, the immigration of government employees with sedentary lifestyles may contribute to the high prevalence of type 2 diabetes in Sejong city. Conversely, Jeju Island, which is geographically isolated and has a distinct population of native peoples, showed the lowest prevalence of type 2 diabetes. The unique environmental and genetic aspects of this area may affect the development of diabetes. Historically, the progenitors of Jeju people had ‘Three Names’ and still more than 15% of total population of Jeju Island have one of these three family names^21^. This suggests that Jeju population have unique genetic characteristics compare to peoples in other regions. Due to its lack of fresh water, paddy farming is only done on a small scale on the island, with the cultivation of cereal crops such as millet, barnyard millet, buckwheat, and barley being the main feature of agriculture. Therefore, the traditional Jeju meal generally consists of multiple grains rather than white rice which is main dish at most of other regions in Korea. In addition, seafood is commonly consumed as a part of the meal. These characteristics of lifestyle might be also able to affect the lower prevalence of diabetes at Jeju Island.

The prevalence of diabetes, when defined as fasting plasma glucose ≥126 mg/dL without codes E11–14 or a prescription of anti-diabetic medication, was 3.1% in the preventive health check-up data, which suggests that more than a quarter of the adult diabetic population either does not know that they have diabetes or do not manage their diabetes at medical institutions in Korea. Although this population has decreased from 3.3% in 2006 to 3.1% in 2013, the rate of decrease is very slight. This indicates that better public planning and measures are needed for undiagnosed or unmanaged diabetic patients even though nationwide preventive health check-ups has been routinely performed in Korea.

In this study, 62.5% and 49.5% of subjects with type 2 diabetes had hypertension and dyslipidemia, which was 3.7-fold and 5-fold higher, respectively, than non-diabetic adults in 2013. The appropriate treatment of hypertension and dyslipidemia in patients with diabetes is critical because of diabetes’ effects on clinical outcomes, including microvascular and macrovascular complications^22^. The rates of controlled hypertension and controlled dyslipidemia(LDL-cholesterol <100 mg/dL) were 75.8% and 45.3%, respectively, which is unacceptable despite improvements during the last several years. These results suggest that intensive interventions and increased clinical attention must be strengthened.

In summary, the present study shows a gradual increase in the number of diabetic patients in Korea during the past 8 years. The fact that the annual incidence of diabetes has not decreased in the 30–39-year-old age group, despite a decreased rate in other age groups, implies the possibility of an increase in patients with longer duration of diabetes and an increased prevalence of diabetes in the future. Furthermore, an increased prevalence of the co-morbid conditions hypertension and dyslipidemia, along with poor rates of control, underlines the need for structured public planning and measures for prevention and proper management of diabetes and co-morbid diseases.

## Methods

### Data sources

This study used the shared National Health Insurance Service database of claims and preventive health check-up data recorded between 1 January 2006 and 31 December 2013.

The shared NHIS claims database(the insurance claims data) contains medical information on all insurance claims for approximately 50 million Koreans, which is more than 99% of the population. This data includes each patient’s encrypted identification number, age, gender, primary diagnosis, secondary diagnoses, date of hospital visits, prescriptions received during inpatient and outpatient visits, hospital admissions, medical and surgical procedures, type of insurance(National Health Insurance or Medical Aid), and medical expenses. Drug information includes the brand name, generic name, prescription date and duration, and route of administration^23^. The diagnoses were coded according to the International Classification of Disease, Tenth Revision(ICD-10). The shared NHIS health check-up database(the preventive health check-up data) contains information for approximately 10 million Koreans who are aged ≥40, employees who are insured, or those who are heads of their household. This data includes height, weight, systolic blood pressure, diastolic blood pressure, fasting plasma glucose and total cholesterol(since 2002); and LDL-cholesterol, HDL-cholesterol and triglyceride levels(since 2009), as well as information on insurance claims.

The study was approved by the Institutional Review Board of the Korean National Institute for Bioethics Policy(P01-201504-21-005). An informed-consent exemption was granted by the board. All methods were performed in accordance with the relevant guidelines and regulations.

### Ascertainment of prevalence and incidence of diabetes

The patients with type 2 diabetes were defined from the insurance claims data as having at least one claim per year for the prescription of anti-diabetic medication under ICD-10 codes E11–14. From the preventive health check-up data, type 2 diabetes was defined as having at least one claim per year for the prescription of anti-diabetic medication under ICD-10 codes E11–14 or having a fasting plasma glucose ≥126 mg/dL without a claim for prescription of anti-diabetic medication under ICD-10 codes E11–14. Anti-diabetic medications included sulfonylureas, metformin, DPP4 inhibitors, thiazolidinediones, alpha-glucosidase inhibitors, meglitinides, and insulins. Type 1 diabetes patients who had claims under ICD-10 code E10 were excluded from this study.

The incidence of type 2 diabetes was estimated by confirming the incident cases that did not meet the criteria of type 2 diabetes(e.g., no claims under ICD-10 codes E11–14 or anti-diabetic medication prescriptions) between 2002 and their diabetes diagnosis. Thus, the incident cases identified after January 1, 2006 had at least a four-year disease-free observational period. The age categorized incidence of T2DM for 8 years(from 2006 to 2013) was also analyzed with NHIS data every year for 8 years as per 1,000 residents of the source population.

### Ascertainment of prevalence of impaired fasting glucose and concomitant co-morbidities

The prevalence of IFG was determined using the preventive health check-up data. Incident cases of impaired fasting glucose were defined as a fasting plasma glucose level of 100–125 mg/dL without either a claim under ICD-10 codes E11–14 or a prescription for anti-diabetic medication, in accordance with the American Diabetes Association(ADA) criteria.

The prevalence of hypertension and dyslipidemia, as well as the rates of controlled disease, were also determined using the preventive health check-up data. Dyslipidemia was defined according to the presence of one or more of the following criteria:(1) the presence of at least one claim per year for the prescription of anti-dyslipidemic medication under ICD-10 code E78,(2) LDL-cholesterol ≥100 mg/dL,(3) triglycerides ≥150 mg/dL, or(4) high density lipoprotein <40 mg/dL in men or <50 mg/dL in women. Subjects with hypertension were defined as having at least one claim per year for the prescription of anti-hypertension medication under ICD-10 codes I10–13 and I15 or having a systolic blood pressure ≥140 mmHg or diastolic blood pressure ≥90 mmHg without a claim for anti-hypertension medication under ICD-10 codes I10–13 and I15. The lipid-controlled standards in diabetic patients were defined as LDL-cholesterol <100 mg/dL, triglycerides <150 mg/dL, and high density lipoprotein ≥40 mg/dL in men and ≥50 mg/dL in women. Controlled hypertension was defined as systolic blood pressure <140 mmHg and diastolic blood pressure <90 mmHg among persons with hypertension.

The prevalence of obesity was also determined using the preventive health check-up data. Obesity defined as body mass index ≥25 kg/m^2^ in accordance with the Asia-Pacific criteria of the WHO guidelines.

### Statistical analysis

The estimates of the annual prevalence rates according to age and gender were obtained by dividing the number of cases of diagnosed type 2 diabetes or IFG identified in the NHIS dataset by the total population enrolled in the NHIS program in a given year. The prevalence rates of the 17 residential areas of South Korea were standardized based on the age of the population in that year. The annual incidence rates of type 2 diabetes were estimated by dividing the number of newly-diagnosed type 2 diabetes cases by the number of insured individuals who did not have type 2 diabetes between 2002 and one year prior to the year in question.

A Poisson distribution was assumed for the annual trends in prevalence and incidence with adjustment for age and gender. Values are expressed as mean ± standard deviation or *n* cases(%). Two-sided p-values ≤ 0.05 were considered statistically significant. All data was analyzed using SAS software version 9.3(SAS Institute, Cary, NC, USA).

## Additional Information

**How to cite this article:** Noh, J. *et al*. Trends in the pervasiveness of type 2 diabetes, impaired fasting glucose and co-morbidities during an 8-year follow-up of nationwide Korean population. *Sci. Rep.* **7**, 46656; doi: 10.1038/srep46656(2017).

**Publisher's note:** Springer Nature remains neutral with regard to jurisdictional claims in published maps and institutional affiliations.

## Supplementary Material

Supplementary Dataset 1

## Figures and Tables

**Table 1:** Prevalence of type 2 diabetes in Korea according to national health insurance claims and national preventive health care database from 2006 to 2013.

	2006	2007	2008	2009	2010	2011	2012	2013
**Health insurance claims database**								
Total population	29,340,486	29,964,692	30,784,595	31,446,745	32,113,334	32,755,158	33,397,816	33,897,232
Age of total population(year)	49.0 ± 13.7	49.4 ± 13.8	49.7 ± 13.8	50.0 ± 13.9	50.3 ± 13.9	50.5 ± 14	50.8 ± 14.1	51.2 ± 14.2
Prevalent cases	1,655,495	1,817,040	1,979,403	2,138,344	2,287,430	2,455,658	2,590,519	2,720,777
Age of prevalent cases(year)	60.7 ± 11.7	61.1 ± 11.8	61.4 ± 11.8	61.7 ± 11.9	62.0 ± 11.9	62.3 ± 11.9	62.6 ± 11.9	62.9 ± 12.0
Prevalence rate(%)	5.6	6.1	6.4	6.8	7.1	7.5	7.8	8.0
**Sex**								
Female	5.3	5.7	6.0	6.3	6.6	6.9	7.1	7.3
Male	6.0	6.5	6.9	7.4	7.7	8.2	8.5	8.8
**Age**								
30–39	0.7	0.8	0.8	0.8	0.8	0.9	0.9	0.9
40–49	3.0	3.1	3.2	3.3	3.3	3.4	3.4	3.5
50–59	7.7	8.0	8.2	8.4	8.5	8.7	8.8	8.9
60–69	13.7	14.3	14.8	15.3	15.8	16.4	16.5	16.6
70–79	15.5	16.7	17.7	18.6	19.5	20.4	21.0	21.5
80–	9.8	11.0	12.1	13.2	14.1	15.1	15.9	16.7
**Preventive health care database**								
Total population	7,229,856	6,642,441	8,284,141	9,186,594	10,225,856	10,456,856	11,015,182	10,610,669
Age of total population(year)	48.6 ± 12.2	49.4 ± 12.6	49.2 ± 12.3	50.1 ± 12.5	50.0 ± 12.5	50.2 ± 12.6	50.5 ± 12.5	50.8 ± 12.7
Age of prevalent cases(year)	56.5 ± 11.7	57.3 ± 11.9	57 ± 11.7	57.8 ± 11.6	58.1 ± 11.6	58.3 ± 11.7	58.6 ± 11.5	59.0 ± 11.7
Prevalence rate(%)	8.4	9.0	8.9	9.9	9.9	10.3	10.5	10.9
**Sex**								
Female	7.3	8.1	7.5	8.5	8.4	8.7	8.8	9.3
Male	9.2	9.6	9.9	11.0	11.2	11.5	11.9	12.2
**Age**								
30–39	2.6	2.7	2.6	2.8	2.6	2.7	2.5	2.6
40–49	5.7	6.1	6.2	6.1	6.0	6.1	6.1	6.2
50–59	10.9	11.1	11.2	11.8	11.8	11.9	12.1	12.4
60–69	16.5	16.6	16.8	18.0	18.6	18.9	19.1	19.6
70–79	18.8	19.3	20.0	20.7	21.8	22.7	23.2	23.8
80–	18.3	18.5	18.9	19.1	20.0	21.2	21.9	22.8

**Table 2:** Incidence of type 2 diabetes in Korea according to national health insurance claims database from 2006 to 2013.

	2006	2007	2008	2009	2010	2011	2012	2013
**Total population**	27,950,337	28,412,787	29,067,525	29,576,957	30,089,468	30,575,216	31,066,304	31,430,044
**Incident Cases**	265,346	265,135	262,333	268,556	263,564	275,716	259,007	253,589
**Age(year)**	58.5±13.0	58.6±13.1	58.1±13.0	58.1±13.0	58.2±13.0	58.2±13.0	58.2±13.0	58.0±13.1
**Incidence rate(%)**	0.95	0.93	0.90	0.91	0.88	0.90	0.83	0.81
**Sex**								
Female	0.84	0.83	0.79	0.80	0.76	0.78	0.71	0.68
Male	1.07	1.04	1.03	1.02	0.99	1.03	0.97	0.94
**Age**								
30–39	0.23	0.23	0.24	0.25	0.24	0.25	0.24	0.25
40–49	0.63	0.61	0.61	0.61	0.58	0.60	0.55	0.54
50–59	1.32	1.27	1.23	1.22	1.16	1.17	1.08	1.03
60–69	2.03	1.94	1.84	1.83	1.75	1.76	1.57	1.47
70–79	2.46	2.37	2.11	2.07	1.97	1.98	1.78	1.65
80–	1.87	1.84	1.60	1.57	1.46	1.47	1.35	1.25

**Table 3:** Prevalence of Impaired fasting glucose according to national preventive health care database from 2006 to 2013.

	2006	2007	2008	2009	2010	2011	2012	2013
**Total population**	7,229,856	6,642,441	8,284,141	9,186,594	10,225,856	10,456,856	11,015,182	10,610,669
**Prevalent Cases**	1,556,670	1,526,109	1,871,249	2,223,858	2,435,394	2,482,891	2,641,361	2,649,471
**Age of prevalent cases(year)**	50.2±12.1	51.1±12.4	50.8±12.0	51.4±12.2	51.6±12.2	51.7±12.3	52.1±12.1	52.4±12.4
**Prevalence rate(%)**	21.5	23.0	22.6	24.2	23.8	23.7	24.0	25.0
**Sex**								
Female	17.9	19.7	18.8	20.6	19.8	19.8	19.8	21.0
Male	24.0	25.3	25.4	27.1	27.1	27.0	27.4	28.2
**Age**								
30–39	17.1	18.1	17.4	19.4	18.3	18.5	18.0	18.9
40–49	21.5	23.1	22.7	23.9	23.4	23.2	23.4	24.1
50–59	24.1	25.3	25.4	26.7	26.5	26.3	26.8	27.8
60–69	24.4	25.9	25.9	26.7	26.7	26.4	26.9	28.0
70–79	24.5	26.2	25.2	25.8	25.9	25.9	26.0	27.3
80–	27.1	28.9	27.3	27.5	28.1	27.6	27.5	28.6
