# Supplementary material for: Trends in the pervasiveness of type 2 diabetes, impaired fasting glucose and co-morbidities during an 8-year-follow-up of nationwide Korean population
Source: Sci Rep. 2017 Apr 20;7:46656. doi: 10.1038/srep46656 (PMC5397969; doi:10.1038/srep46656)
Supplement: Supplementary Dataset 1 [file srep46656-s1.doc]

**Trends in the pervasiveness of type 2 diabetes, impaired fasting glucose and co-morbidities during an 8-year-follow-up of nationwide Korean population**

Junghyun Noh^1^, Kyung-Do Han^2^, Seung-Hyun Ko^3^, Kyung Soo Ko^4^, Cheol-Young Park^5^

^1^Department of Internal Medicine, Inje University Ilsan Paik Hospital, Goyang, Republic of Korea.

E-mail: jhnoh@paik.ac.kr

^2^Department of Medical Statistics, Catholic University College of Medicine, Seoul, South Korea.

E-mail: hkd917@naver.com

^3^Department of Internal Medicine, St. Vincent's Hospital, The Catholic University of Korea, Seoul, Korea.

E-mail: kosh@catholic.ac.kr

^4^Department of Internal Medicine, Cardiovascular and Metabolic Disease Center, Inje University Sanggye Paik Hospital, Seoul, Republic of Korea.

E-mail: kskomd@paik.ac.kr

^5^Department of Internal Medicine, Kangbuk Samsung Hospital, Sungkyunkwan University School of Medicine, Seoul, Republic of Korea.

SUPPLEMENTARY DATA

Table 1. Incidence of type 2 diabetes for 8 years in Korea according to national health insurance claims database.

|  | Total | | | FEMALE | | | MALE | | |
| --- | --- | --- | --- | --- | --- | --- | --- | --- | --- |
| Age (year) | number of event | Person-Years | Incidence rate (per 1000) | number of event | Person-Years | Incidence rate (per 1000) | number of event | Person-Years | Incidence rate (per 1000) |
| 30-39 | 211,856 | 69,773,671 | 3.04 | 63017 | 34,284,550 | 1.84 | 148839 | 35,489,121 | 4.19 |
| 40-49 | 475,327 | 65,437,443 | 7.26 | 158931 | 32,332,982 | 4.92 | 316396 | 33,104,461 | 9.56 |
| 50-59 | 519,574 | 40,996,247 | 12.67 | 221044 | 20,835,913 | 10.61 | 298530 | 20,160,334 | 14.81 |
| 60-69 | 437,372 | 25,761,709 | 16.98 | 228450 | 13,761,492 | 16.60 | 208922 | 12,000,217 | 17.41 |
| 70-79 | 235,086 | 14,287,888 | 16.45 | 145449 | 8,658,593 | 16.80 | 89637 | 5,629,295 | 15.92 |
| 80- | 52,189 | 5,540,367 | 9.42 | 36135 | 3,889,304 | 9.29 | 16054 | 1,651,062 | 9.72 |

Table 2. Control rate of hypertension and dyslipidemia in type 2 diabetes from 2006 to 2013.

|  | **2006** | **2007** | **2008** | **2009** | **2010** | **2011** | **2012** | **2013** |
| --- | --- | --- | --- | --- | --- | --- | --- | --- |
| **Hypertension Control Rate (%)** | | | | | | | | |
| SBP < 140 mmHg | 71.1 | 73.2 | 74.9 | 77.0 | 77.2 | 77.4 | 78.1 | 79.3 |
| DBP < 90 mmHg | 77.4 | 79.7 | 81.4 | 83.1 | 83.4 | 84.1 | 84.6 | 85.9 |
| SBP < 140 and DBP < 90 mmHg | 66.7 | 69.2 | 71.2 | 73.5 | 73.6 | 73.8 | 74.5 | 75.8 |
| **Dyslipidemia Control Rate (%)** | | | | | | | | |
| LDL-C < 100 mg/dL |  |  |  | 40.2 | 40.8 | 42.9 | 44.0 | 45.3 |
| Triglyceride < 150 mg/dL |  |  |  | 52.2 | 52.6 | 53.9 | 55.0 | 55.2 |
| HDL-C ≥ 40 in men and ≥ 50 mg/dL in women |  |  |  | 70.5 | 71.0 | 70.2 | 69.8 | 68.4 |
| LDL-C <100 and TG < 150 and HDL-C ≥ 40 in men and ≥ 50 mg/dL in women |  |  |  | 15.3 | 16.6 | 17.7 | 18.4 | 18.8 |


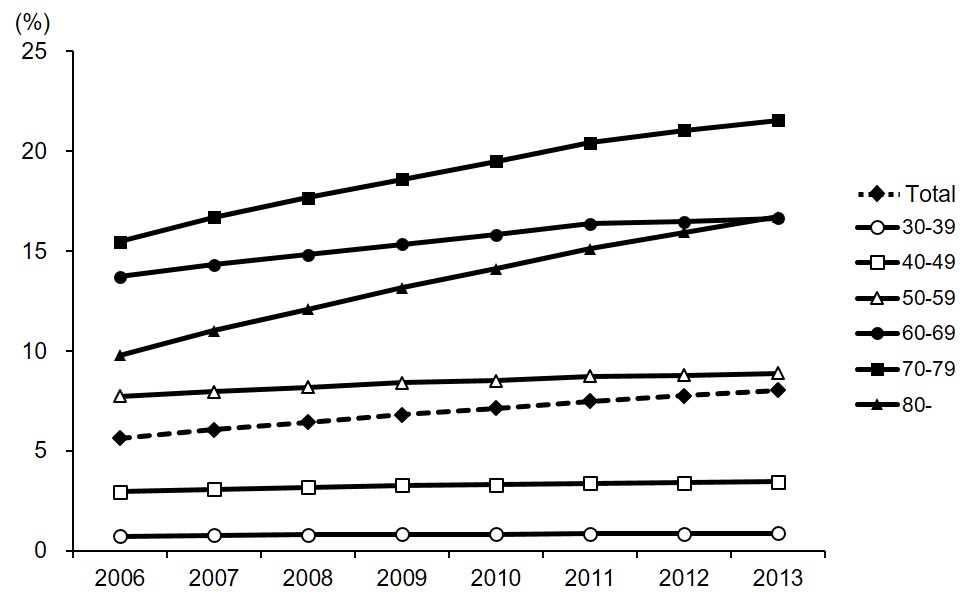


Supplementary figure 1.


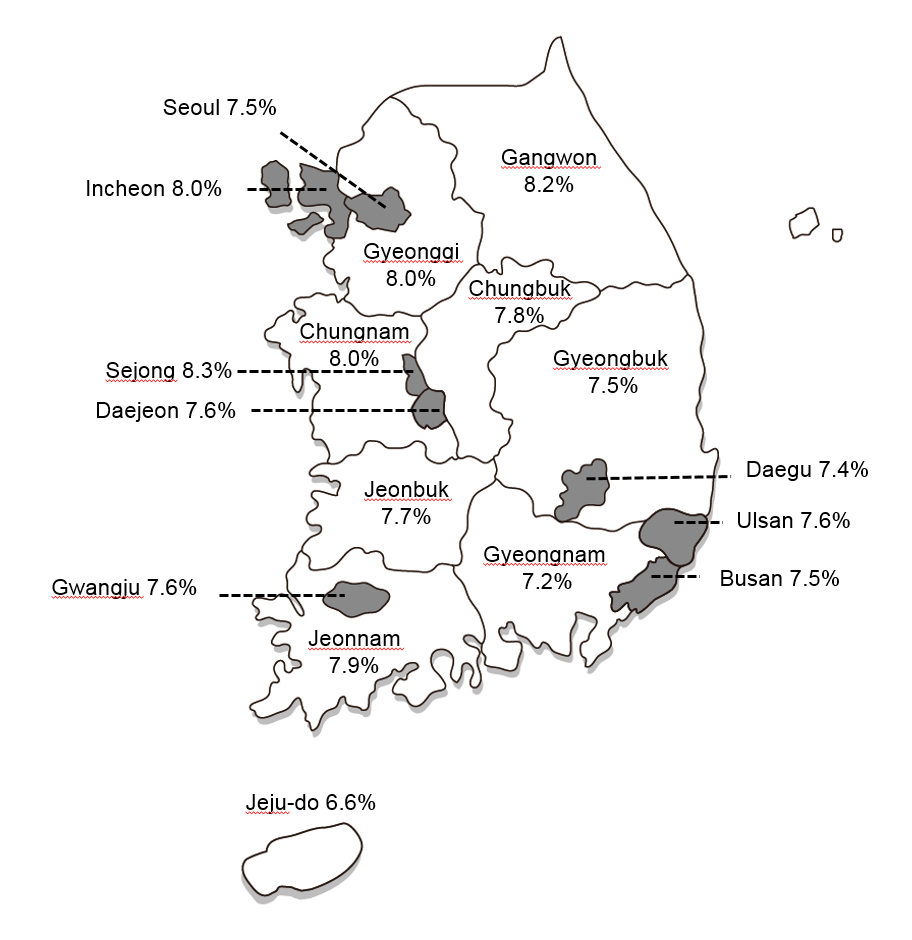


Supplementary figure 2.


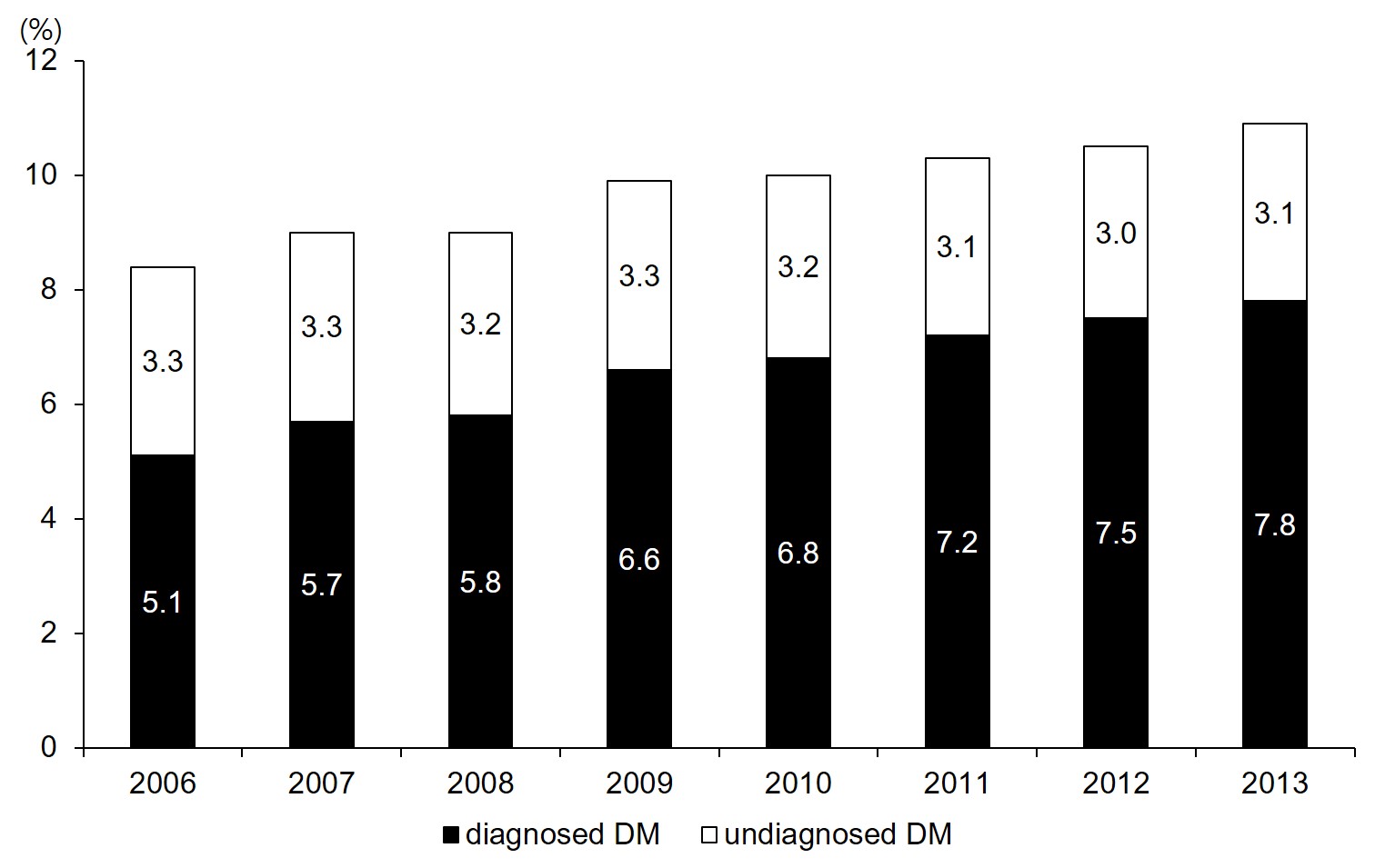


Supplementary figure 3.

SUPPLEMENTARY FIGURE LEGENDS

Supplementary Figure. 1 Time trends in the prevalence of type 2 diabetes stratified by age according to national health insurance claims database.

Supplementary Figure 2. Age-standardized prevalence of diabetes according to region in 2013. The map was created using adobe illustrator CS3 software. https://www.adobe.com/

Supplementary Figure 3. Prevalence of diagnosed and undiagnosed diabetes according to national preventive health care database from 2006 to 2013.
